# Finding meanings in Late onset Post Traumatic Stress Disorder – a review of the literature

**DOI:** 10.1192/j.eurpsy.2022.1738

**Published:** 2022-09-01

**Authors:** B. Ramos, I. Soares Da Costa, A. Elias De Sousa, F. Andrade, F. Santos Martins

**Affiliations:** Centro Hospitalar Universitário São João, Psychiatry And Mental Health Department, Porto, Portugal

**Keywords:** Late Onset Post Traumatic Stress Disorder, triggers, prevention, Trauma

## Abstract

**Introduction:**

About a decade ago, the idea of a Late-Onset Post Traumatic Stress Disorder (LO-PTSD) emerged, in order to characterize the later-life emergence of symptoms related to early-life warzone trauma among aging combat Veterans.

**Objectives:**

This paper provides a review of the changes happened during the onset of a late form of PTSD and how can mental health professionals intervene.

**Methods:**

Review of the literature from 2015 to present, using search engines such as Pubmed and Google Schoolar, using the following keywords: Late-Onset Post Traumatic Stress Disorder, triggers, prevention, intervention

**Results:**

At first, there was hypothesized that aging-related challenges (role transition and loss, death of family members and friends, physical and cognitive decline) might lead to increased reminiscence, and possibly distress, among Veterans who had previously dealt successfully with earlier traumatic events. However, recent studies have proposed that in later life many combat Veterans confront and rework their wartime memories in an effort to find meaning and build coherence. Through reminiscence, life review, and wrestling with issues such as integrity versus despair, they intentionally reengage with experiences they avoided or managed successfully earlier in life, perhaps without resolution or integration. This process can lead positively to personal growth or negatively to increased symptomatology.

**Conclusions:**

Therefore the role of preventive intervention in enhancing positive outcomes for Veterans who reengage with their wartime memories in later life should be reconsidered.

**Disclosure:**

No significant relationships.

